# Suggestions for Culturally Adapted Interventions to Treat Substance Use in Immigrants

**DOI:** 10.7759/cureus.113006

**Published:** 2026-07-20

**Authors:** Dhiya Ram, Kathryn N Margiotta, Pujita Julakanti, Raymond L Ownby

**Affiliations:** 1 Psychiatry and Behavioral Sciences, Nova Southeastern University Dr. Kiran C. Patel College of Osteopathic Medicine, Davie, USA; 2 College of Osteopathic Medicine, Nova Southeastern University Dr. Kiran C. Patel College of Osteopathic Medicine, Davie, USA; 3 Internal Medicine, Nova Southeastern University Dr. Kiran C. Patel College of Osteopathic Medicine, Fort Lauderdale, USA

**Keywords:** addiction psychiatry, cultural competence, immigrant health, substance use disorder (sud), treatment choices

## Abstract

Immigrant populations experience unique sociocultural, structural, and linguistic barriers that shape substance use disorder (SUD) risk, treatment engagement, and outcomes. Conventional treatment models often fail to account for these complexities, contributing to disparities in care. This systematic review evaluates specific culturally adapted interventions for SUD treatment in immigrant populations across diverse sociocultural and regional contexts, identifying which strategies improve engagement and outcomes, and highlighting gaps in their development and evaluation. A systematic search of EMBASE, Ovid MEDLINE, and Web of Science identified studies examining addiction treatment in immigrant populations. After screening and eligibility assessment, 19 studies were included. Data, including the population and treatment or intervention studied, major findings, and relevant remaining gaps, were extracted for assessment. We extracted data from 19 studies out of 254 screened. Among the included studies, culturally adapted interventions fell into one of several key domains: language concordance, structural barrier reduction, stigma-targeted approaches, culturally rooted practices, increasing provider cultural competence, social support integration, and treatment of co-occurring mental health disorders. Interventions that addressed linguistic barriers and were culturally tailored to the immigrant population were most consistently associated with improved engagement and retention, while structural and stigma-related barriers remained major determinants of treatment access. Effective interventions for immigrants with SUDs require culturally informed, person-centered, and community-oriented approaches that account for the unique lived experiences of immigrant populations. Future research should prioritize expanding culturally tailored interventions across diverse sociocultural and regional contexts to better capture the heterogeneity of immigrant experiences, along with further clinical research to evaluate their effectiveness and real-world applicability.

## Introduction and background

Substance use disorders (SUDs) represent a major public health concern, with patterns of use and treatment response differing significantly across population groups. Among immigrant populations in particular, these differences are driven by distinct psychosocial, structural, and cultural influences that shape both risk and engagement with care in ways not observed in non-immigrant groups. SUDs are a clinical condition involving the problematic pattern of alcohol or drug use that results in significant impairment or distress, including loss of control over use and adverse social, occupational, or health consequences. Understanding risk factors for the development of SUDs in immigrant populations may help improve prevention, early recognition, and treatment outcomes. Existing research has highlighted that treatment engagement is diminished when individuals feel they are not recognized or valued as whole persons within standard care settings, particularly when their identity as an immigrant, immigration history, and lived experiences are overlooked [1]. The effectiveness of SUD interventions in immigrant populations depends largely on how closely care aligns with patients’ cultural values, perceptions of the healthcare system, and understanding of substance misuse [2,3]. Treatment of SUD in immigrants requires a more nuanced, holistic approach that is culturally and contextually attuned to patients’ lived experiences but that remains insufficiently addressed in current treatment models.

Social stressors such as racial prejudice, discrimination, and social isolation are well-established contributors to the development of mental illness and unhealthy coping behaviors such as substance misuse [4], thereby increasing both vulnerability to and the complexity of co-occurring mental health conditions and SUDs within this population. Immigrants face significant systemic barriers that further limit access to and engagement in care, particularly for SUDs [5]. Both documented and undocumented immigrants have been reported to avoid seeking treatment or reporting substance use due to fear of repercussions, such as deportation or the impact of an SUD diagnosis on their citizenship application [5]. When seeking permanent residence in the US, for instance, non-citizens must report any history of an SUD, which conflicts with the requirement for “good moral character” rather than being regarded as a medical condition, thus perpetuating the stigma of SUDs on both a cultural and judicial level [5]. Systemic barriers to accessing care for SUDs among immigrants not only limit individuals’ ability to obtain needed treatment but likely distort available data on substance use rates and the effectiveness of interventions within these populations.

Risk and protective factors among immigrants are shaped by unique dynamics, including the interplay between family and community support and the effects of acculturation, making them essential to understand for effective prevention, education, and treatment. Greater integration into social sectors where drug culture is more prevalent and normalized has been shown to increase the likelihood of substance use, though this effect varies across racial and ethnic groups and depends on how assimilation impacts family dynamics [6,7]. Family connectedness and parental involvement may potentially offset the impacts of affiliation with perceived substance-using peers [1,8], suggesting that family strengthening programs may represent one method of preventing and addressing SUDs in certain contexts. Other studies have revealed that immigrants with SUDs are more likely to seek support from community centers or trusted friends and family rather than healthcare providers who may not align with their cultural perspectives on addiction and its social impact [9]. Approaches to treatment of immigrants with addiction that incorporate family systems, schools, and community-based settings may be particularly impactful in strengthening early prevention and intervention efforts within immigrant populations [6]. Consistent with the effect of acculturation on the development of SUDs, white immigrants, who are less likely to face racial prejudice as a barrier to social integration, typically experience significantly higher rates of substance misuse than their racial and ethnic counterparts [6,10]. In contrast, lower observed rates of substance misuse among non-white immigrants may contribute to the assumption that they are at reduced risk for developing SUDs, which in turn may partially explain why immigrants of color remain underrepresented in research and underserved in SUD treatment [10].

Despite these insights, there remains a large gap in research evaluating how to best translate what we know about immigrants’ perceptions of and response to treatment for SUDs into effective, culturally adapted interventions. Disparities in treatment of immigrants with SUDs are not solely driven by access but by the degree to which care is culturally responsive, person-centered, and structurally attuned to the realities of migration and marginalization. Immigrant populations remain underrepresented in research on SUDs, and existing treatment models often fail to account for the heterogeneity of experiences that shape substance use and recovery. There is limited evidence guiding the development and implementation of tailored interventions that can effectively address the unique needs of immigrants with SUDs. This systematic review seeks to answer: what culturally adapted interventions for SUD treatment have been implemented in immigrant populations, and what is known about their impact on treatment engagement, retention, and outcomes? Accordingly, this systematic review aims to characterize existing culturally adapted approaches to SUD treatment in immigrant populations and to identify effective strategies and critical areas for future research.

## Review

Methods

An initial literature search was conducted on Google Scholar to gain a brief overview of the topic, after which certain keywords were sought from relevant articles. With addiction being the pathology of interest, the keyword “addiction” was searched across abstracts, keywords, and titles, alongside second-level keywords, including “addictive behavior” OR "addictive behaviour” OR “addictive dependency” OR “addictive disorder” OR “behavior, addictive” OR “behaviour, addictive” OR “chemical dependence” OR “usage disorder.” Next, “immigrant” was searched across abstracts, keywords, and titles. Finally, the keyword “therapy” was searched across abstracts, keywords, and titles, alongside second-level keywords, including “combination therapy” OR “disease therapy” OR “disease treatment” OR “diseases treatment” OR “disorder treatment” OR “disorders treatment” OR “efficacy, therapeutic” OR “illness treatment” OR “medical therapy” OR “medical treatment” OR “multiple therapy” OR “polytherapy” OR “somatotherapy” OR “therapeutic action” OR “therapeutic efficacy” OR “therapeutic intervention” OR “therapeutic trial” OR “therapeutic trials” OR “therapeutics” OR “therapy, medical” OR “treatment effectiveness” OR “treatment efficacy” OR “treatment, medical.” Articles from these three searches were linked via the Boolean operator AND. The resulting articles were filtered by English language and date range 2016-2026. The search strategy was replicated across three databases, namely EMBASE, Ovid MEDLINE, and Web of Science on February 12, 2026. The initial search yielded 320 articles.

**Figure 1:**
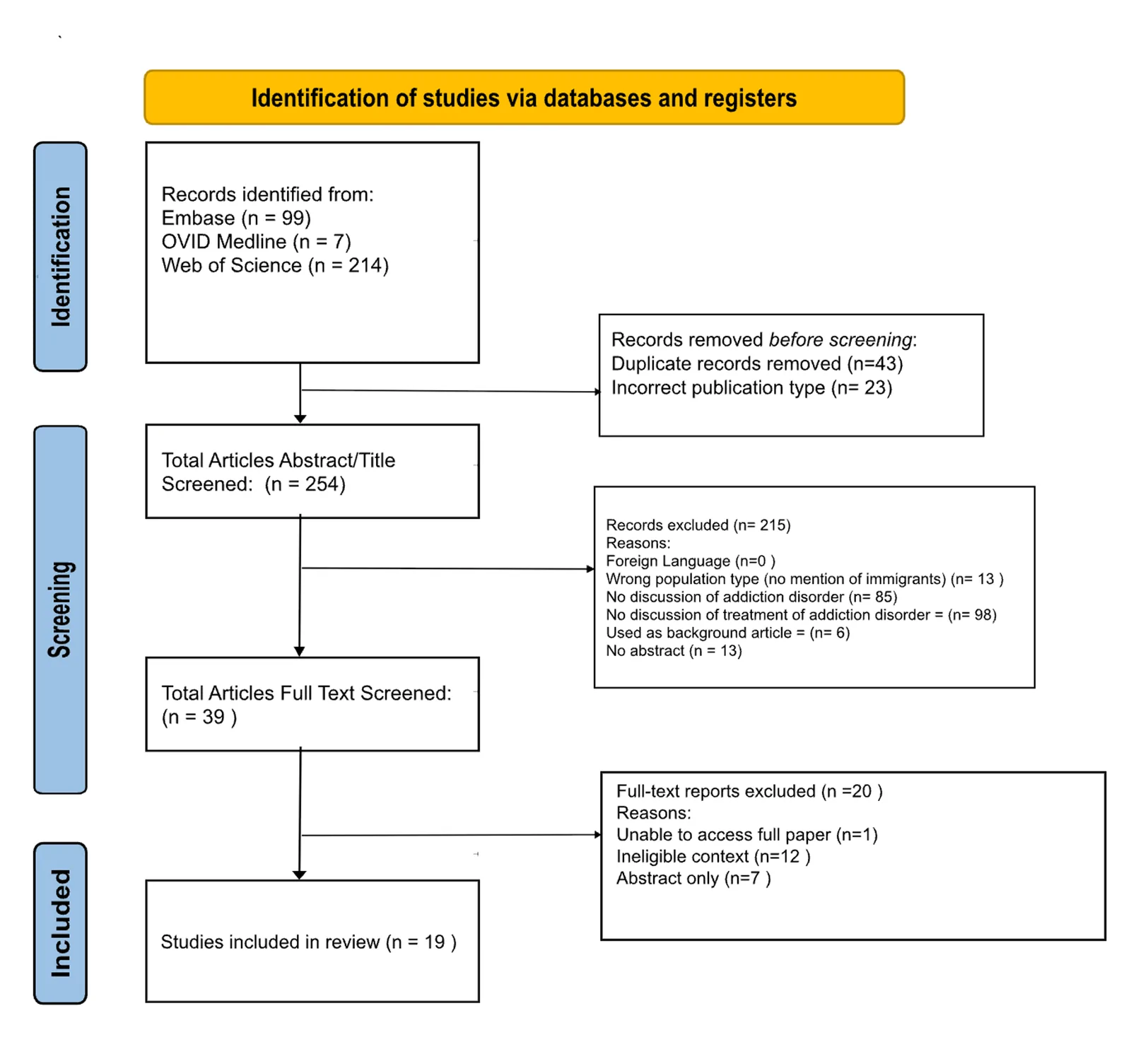
Preferred Reporting Systems for Systematic Reviews and Meta-Analyses (PRISMA) flowchart of the selection procedure

Following import of the 320 articles into EndNote 21 (Clarivate, Philadelphia, PA), 43 duplicate records were removed. The remaining articles were then imported into Rayyan [11] (Rayyan Systems Inc., Cambridge, MA, US) for screening and organization. Before Tier 1 screening, 23 articles were immediately excluded because they did not meet the required publication type criteria for systematic reviews. During Tier 1 and Tier 2 screening, three contributing authors independently reviewed each article’s title, abstract, and full text, as appropriate to the stage of review, to determine inclusion or exclusion based on predefined eligibility criteria. The remaining 254 articles underwent Tier 1 title and abstract screening, after which 215 were excluded, leaving 39 articles. Twenty more articles were excluded after full-text screening, leaving 19 articles for inclusion in the scoping review. The Preferred Reporting Items for Systematic Reviews and Meta-Analyses (PRISMA) flowchart details the study’s article screening and selection process (Figure 1) [12].

Data were extrapolated from the final 19 papers and charted independently by each of the three reviewers using an Excel (Microsoft Corp., Redmond, WA, USA) data extraction form. The variables used in the form included aspects of study design, methodology, and outcomes. These variables were author, year, title, journal, study design, population studied, major findings, and relevant remaining gaps. Artificial intelligence (AI) or any other form of automated assistance was not used at any stage of this review. All processes, including screening, selection, and analysis, were conducted manually by the authors of this review.

Results

Data were extracted from the final 19 papers and charted independently by each of the three reviewers. The variables used in the form included aspects of the study design and suggested intervention. Table 1 presents the 19 articles analyzed for study design, suggested interventions, and the number of subjects in the selected studies [13-31].

**Table 1:** Analysis of selected studies SUD, substance use disorder.

Study	Study design	Suggested intervention	Number of subjects	Population studied
Alegría et al., 2019 [13]	Randomized clinical trial	Bridging the language gap, addressing structural barriers, and treating the underlying primary mental health disorder	341	Latino immigrants in Boston, Massachusetts, and Madrid and Barcelona, Spain
Cox et al., 2022 [28]	Longitudinal study	Incorporating social support	265	Latino early adolescents, including first-generation immigrants
Falgas-Bagué et al., 2019 [14]	Randomized clinical trial	Bridging the language gap and reducing social stigma	341	Latino immigrants in Boston, Massachusetts, and Madrid and Barcelona, Spain
Garcia et al., 2021 [26]	Qualitative study	Using specific culturally rooted traditions	15	Mexican immigrant male farmworkers in southeastern Pennsylvania
Garcia et al., 2017 [15]	Ethnographic qualitative study	Bridging the language gap and using specific culturally rooted traditions	50	Latino males in a mid-size city in Northern California, including undocumented immigrants
Guan et al., 2020 [20]	Observational cross-sectional analysis	Addressing structural barriers	340	Foreign-born Chinese and Vietnamese male daily smokers
Guerrero et al., 2017 [21]	Observational study	Addressing structural barriers	15,412	Adults in publicly funded SUD treatment programs in Los Angeles County, including 46.3% Mexican Americans
Hassan et al., 2020 [23]	Mixed methods convergent design	Reducing social stigma and using specific culturally rooted traditions	93	Muslim adults recruited from local mosques in Toronto
Kour et al., 2021 [16]	Qualitative study	Bridging the language gap and increasing physician/societal cultural competence	19	Norwegians of ethnic and immigrant origin
Lee and Sherman, 2024 [24]	Case study	Reducing social stigma	1	47-year-old Latinx male with alcohol use disorder
McCabe, 2021 [22]	Cross-sectional	Addressing structural barriers	391	Adult Latinx immigrants in North Carolina
Ornelas, 2019 [17]	Randomized clinical trial	Bridging the language gap and using specific culturally rooted traditions	121	Latino immigrant men at day labor worker center in Seattle meeting criteria for alcohol use disorder
Sanchez et al., 2020 [29]	Retrospective cohort study	Incorporating social support	177	Adults aged 18-34 years in Miami-Dade County, Florida, having immigrated from a Latin American country within the past year.
Seid et al., 2024 [18]	Retrospective study	Bridging the language gap, addressing structural barriers, and reducing social stigma	49,330	Individuals (aged 18-90 years) incarcerated in Danish prisons between 2008 and 2018
Sofer et al., 2018 [30]	Retrospective study	Treating the underlying primary mental health disorder	323	All patients admitted to a dual diagnosis unit in Israel, including ex-USSR- and Ethiopian-born immigrants
Stylianopoulos et al., 2024 [19]	Delphi study	Bridging the language gap and increasing physician/societal cultural competence	N/a	Immigrants in Germany
van Selm et al., 2023 [27]	Delphi study	Increasing physician/societal cultural competence	N/a	N/a
Villongco et al., 2025 [25]	Observational cross-sectional analysis	Reducing social stigma	203,295	Pooled data from 2015 to 2019 National Survey of Drug Use and Health (NSDUH) in individuals aged ≥18 years, including Asian American immigrants
Wada et al., 2017 [31]	Cross-sectional	Other	1899	English- and Spanish-speaking patients in a large, public safety-net hospital in Los Angeles County, of whom 68% were foreign-born immigrants

**Figure 2:**
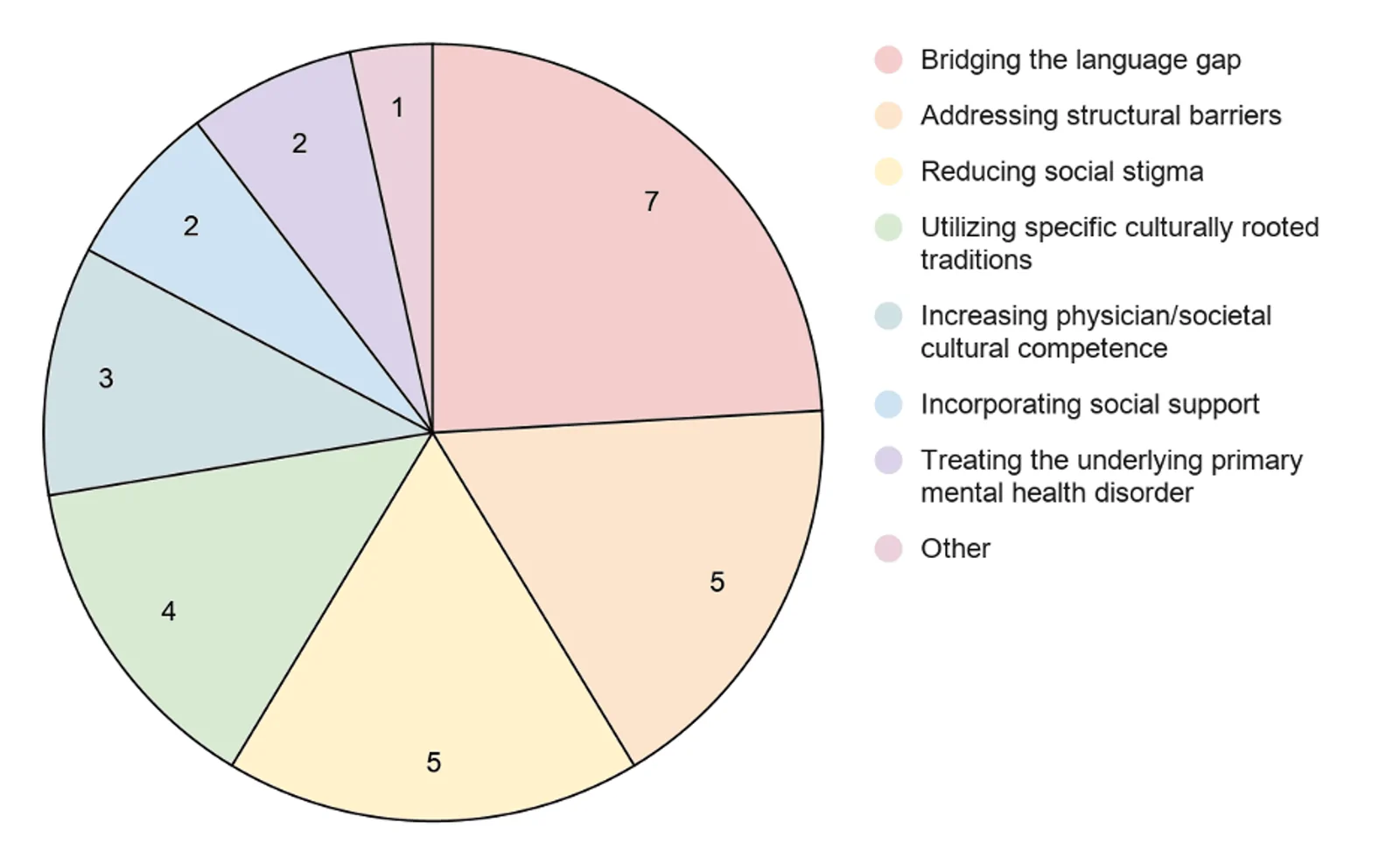
This pie chart summarizes the findings of the studies recorded in Table 1 and groups the suggested interventions by the number of papers identified in this review.

Figure 2 shows a pie chart of the most prevalent suggested interventions to address SUD in immigrant populations based on the number of papers found in this scoping review of recent literature: bridging the language gap with seven articles, addressing structural barriers with five articles, reducing social stigma with four articles, utilizing specific culturally rooted traditions with four articles, increasing physician/societal cultural competence with three articles, incorporating social support with two articles, treating the underlying primary mental health disorder with two articles, and other interventions with two articles [13-31].

Bridging the Language Gap

Table 2 shows the pertinent findings of articles that discuss bridging the language gap as a modality to improve treatment for immigrant populations [13-19].

**Table 2:** Pertinent findings of studies suggesting bridging the language gap.

Study	Pertinent findings
Alegría et al., 2019 [13]	Linguistic matching likely improved retention and acceptability of Integrated Intervention for Dual Problems and Early Action (IIDEA).
Falgas-Bagué et al., 2019 [14]	"Fear of not knowing how to communicate problems because of language barriers” was communicated as a barrier to care by study participants.
Garcia et al., 2017 [15]	Spanish language use and a familiar culture were reported as major reasons that the men seek out and remain in anexos.
Kour et al., 2021 [16]	Participants described how difficulties due to language barriers led to miscommunication, limiting diagnosis and treatment, and problems in group therapy sessions, which lowered treatment engagement among immigrants with co-occurring substance use disorder (SUD) and mental health disorders.
Ornelas, 2019 [17]	Cultural adaptations of screening and brief intervention (SBI) in primary care for reducing unhealthy drinking included offering referral to low-cost Spanish-speaking services.
Seid et al., 2024 [18]	Identified language barriers as an explanation for why people who are incarcerated with an immigrant background were less likely than Danish natives to participate in prison-based treatment for SUD.
Stylianopoulos et al., 2024 [19]	Various aspects of language mediation and multilingual materials were identified as good practices for better communication and proper treatment.

Language barriers have been identified by several papers in this review as a major gap to bridge to provide culturally competent care to immigrant populations [13-19]. Subjectively, participants of multiple studies have identified that difficulties due to language barriers have resulted in miscommunication, fear of not knowing how to communicate problems, and difficulties participating in group therapy sessions. These challenges have resulted in lowered treatment engagement for SUD [14,16,18]. In fact, relatability in terms of linguistics has been shown to increase non-English-speaking participants’ engagement with treatment for SUD. For example, anexos are culturally and linguistically specific recovery houses with a long history in Latino communities across the United States. It has been shown that Spanish language use in anexos is a major reason why Latino males seek out and remain in anexos for completion of SUD treatment [15]. A study assessing the effectiveness of Integrated Intervention for Dual Problems and Early Action (IIDEA) therapy for Latino immigrants with SUD and co-occurring mental health symptoms suggested that linguistic matching improved retention and acceptability of the treatment among participants [13].

In terms of clear linguistic communication, both access to and the implementation of language mediation, as well as the professionalism of the mediators, play a role in bridging the language gap [19]. One study using the Delphi process to identify strategies of good practice in reaching and treating refugees in addiction care suggested that clear linguistic communication serves two purposes: transmission of correct clinical information and serving as a signal of openness and welcome. As a result, seven strategies were identified as good practice per this study: addressing people in their mother tongue as a gesture of welcome, nationwide implementation of language mediation in addiction support facilities, ensuring funding of language mediators, availability of fast and low-threshold language mediators, professionalism of the language mediators employed, supervision for the language mediators, and multilingualism of documents of the facility process [19].

Addressing Structural Barriers

Table 3 shows the pertinent findings of articles that discuss addressing structural barriers as a modality to improve treatment for immigrant populations [13,15,18,20-22].

**Table 3:** Pertinent findings of studies suggesting addressing structural barriers. aOR, adjusted odds ratio; CI, confidence interval; IIDEA, Integrated Intervention for Dual Problems and Early Action; OR, odds ratio.

Study	Pertinent findings
Alegría et al., 2019 [13]	In-person patients were more likely to experience improvement than those who received IIDEA therapy over telephone.
Garcia et al., 2017 [15]	Anexos function as an entire continuum of care for undocumented immigrants who are excluded from mainstream treatment due to cost, insurance, or immigration status.
Guan et al., 2020 [20]	Seeing a doctor in the past year was associated with significantly more quit attempts.
Guerrero et al., 2017 [21]	The interaction between Mexican Americans and programs’ accepting Medicaid payment was statistically significant (OR: 1.284; 95% CI: 1.008, 1.637), meaning that improvements in treatment completion for those treated in programs accepting Medicaid were greater.
McCabe, 2021 [22]	Acculturative stress was positively associated with alcohol use severity, drug use severity, and any drug use.
Seid et al., 2024 [18]	Incarcerated individuals with immigrant backgrounds were also less likely to receive treatment (aOR: 0.92, CI: 0.86-0.99) compared with natives, for reasons including reduced awareness of their right to receive treatment in prison.

Some studies in this review have identified certain systemic structural barriers that impede SUD care for immigrant populations [13,15,18,20-22]. Systemic stressors such as acculturative stress were shown to be positively associated with alcohol use severity, drug use severity, and any drug use in Latinx immigrants in the United States [22]. Often, as a result of these systemic stressors, immigrant patients are unlikely to attend their medical appointments. However, studies in this review show that seeing a doctor in person or within the past year resulted in significantly more quit attempts and an increased likelihood of experiencing improvement [13,20].

Another structural barrier that has been identified as a hindrance in treatment for immigrants is the programs’ willingness to accept Medicaid. One study showed that Mexican Americans’ interactions with programs accepting Medicaid payment were statistically significant (OR: 1.284; 95% CI: 1.008, 1.637) [21]. In fact, addressing this barrier is one of the strengths of settings like anexos, as they were shown to function as an entire continuum of care for undocumented immigrants who are excluded from mainstream treatment due to cost, insurance, or immigration status [15].

In addition to the above-mentioned systemic structural barriers, this review identifies immigrants’ reduced awareness of their rights. For example, Seid et al. identified that in a Danish prison, incarcerated individuals with immigrant backgrounds were less likely to receive treatment for SUD compared with their native counterparts for reasons including reduced awareness of their right to receive treatment while in prison [18].

Reducing Social Stigma

Table 4 shows the pertinent findings of articles that discuss reducing social stigma as a modality to improve treatment for immigrant populations [14,18,23-25].

**Table 4:** Pertinent findings of studies suggesting reducing social stigma. aOR, adjusted odds ratio; CI, confidence interval.

Study	Pertinent findings
Falgas-Bagué et al., 2019 [14]	Mistrust in previous behavioral health treatments was the reported barrier significantly associated with completion of the program after adjusting for clinical, psychosocial, and cultural factors, with those expressing mistrust in previous treatments showing higher rates of completion than those who did not report this barrier.
Hassan et al., 2020 [23]	More than 50% of participants indicated that the primary barrier to accessing services was stigma.
Lee and Sherman, 2024 [24]	Augmenting motivational interviewing with self-affirmation theory may be particularly impactful for individuals who have experienced stigma.
Seid et al., 2024 [18]	Incarcerated individuals with immigrant backgrounds were also less likely to receive treatment (aOR: 0.92, CI: 0.86-0.99) compared with natives for reasons including cultural stigma.
Villongco et al., 2025 [25]	Asian Americans with alcohol and substance use disorder were more likely to use less formal and a combination of both formal and informal treatments when compared with participants who were White, Black, and Hispanic (p<0.05, 95% CI: 0.03-0.79).

Sociocultural stigma concerning attitudes toward and perception of SUD and its treatment was identified as another factor to address in order to appropriately treat immigrants in need of substance use treatment [14,18,23-25]. In one study, more than 50% of the participants subjectively shared that the primary barrier to accessing mental health services among Muslim-Canadians was stigma. In fact, following a 90-minute interactive faith-based psychoeducational addiction seminar, there was a significant increase in the participants’ self-reported knowledge (p<0.001) and an increase in willingness to seek help from a mental health professional (p<0.03) [23]. Similarly, incarcerated individuals with an immigrant background in Danish prisons were also less likely to receive treatment (aOR: 0.92, CI: 0.86-0.99) compared with natives for reasons including cultural stigma. Sociocultural stigma further explains why Asian Americans with alcohol and SUD were more likely to use less formal or combinations of formal and informal treatment when compared with participants of other ethnic backgrounds (p<0.05) [25]. One proposed intervention for addressing social stigma with SUD treatment was augmenting motivational interviewing with self-affirmation theory [24].

Additionally, some stigma identified in immigrant populations does not have sociocultural roots but was developed after prior behavioral health treatment. However, some treatment modalities have been shown to be effective in such populations with developed mistrust. One such treatment modality is IIDEA; its implementation among folks with mistrust in prior treatment showed significant completion of the current treatment course (aOR: 3.79, CI: 1.49-9.66) [14].

Utilizing Specific Culturally Rooted Traditions

Table 5 shows the pertinent findings of articles that discuss utilizing specific culturally rooted traditions as a modality to improve treatment for immigrant populations [15,17,23,26].

**Table 5:** Pertinent findings of studies suggesting utilizing specific culturally rooted traditions.

Study	Pertinent findings
Garcia et al., 2021 [26]	Juramentos were made by study participants. Nearly all 15 of the farmworkers, except for two, remained abstinent during the abstinence period and did not make another until they saw it necessary, either to fend off the temptation to drink or to stop drinking after resuming. The two who broke their pledge made another Juramento and fulfilled it.
Garcia et al., 2017 [15]	Anexos function as an entire continuum of care for undocumented immigrants who are excluded from mainstream treatment due to cost, insurance, or immigration status.
Hassan et al., 2020 [23]	Psychoeducation programs that combine Islamic principles and medical knowledge can successfully reduce stigma in Muslim populations.
Ornelas, 2019 [17]	There were larger and more consistent changes in drinking quantity and frequency in the intervention group, which utilized culturally adapted interventions using an established Vida PURA framework.

Certain studies have identified culturally specific traditions that have been effective in SUD treatment in certain immigrant populations [15,17,23,26]. One study identified Juramentos, translating to mean oath or pledge, as an effective way to encourage abstinence among Hispanic men in the United States. Per this study, nearly all 15 of the participants, except for two, remained abstinent following their first Juramento. The two who broke the pledge made another Juramento and fulfilled it [26]. Another effective treatment structure for Hispanic folks in the United States is anexos, serving as culturally and linguistically specific recovery houses that offer a continuum of care for undocumented immigrants who are excluded from mainstream treatment due to factors such as cost, insurance, or immigration status [15]. In terms of Hispanic immigrants in the United States, another intervention that has been shown to lead to larger and more consistent changes with alcohol use utilized culturally adapted interventions using an established Vida PURA framework [17].

In terms of non-Hispanic American populations, only one study was identified by the review to provide population-specific forms of treatment. Hassan et al. showed that psychoeducation programs that combine both Islamic principles and medical knowledge can successfully reduce stigma and encourage treatment engagement in Muslim populations [23].

Increasing Physician/Societal Cultural Competence

Table 6 shows the pertinent findings of articles that discuss increasing physician and societal cultural competence as a modality to improve treatment for immigrant populations [16,19,27].

**Table 6:** Pertinent findings of studies suggesting increasing physician/societal cultural competence.

Study	Pertinent findings
Kour et al., 2021 [16]	Participants described how the lack of cultural competence caused misunderstandings of what is socially and culturally acceptable behavior for immigrant patients and hence decreased treatment engagement.
Stylianopoulos et al., 2024 [19]	This Delphi study identified some good practices in reaching and treating refugees in addiction care, including “understanding and acceptance of substance use as a coping strategy.”
van Selm et al., 2023 [27]	This Delphi study identified some strategies to improve healthcare access for migrants who use drugs, including “campaigns and interventions to reduce all forms of stigma, discrimination, and racism, targeting both service providers and migrants who use drugs themselves.”

In addition to factors such as language gap, studies have identified physician and societal cultural competence as a factor that hinders immigrant populations from seeking SUD care [16,19,27]. In one study identified by this review, patients subjectively described how the lack of cultural competence among providers caused misunderstandings of what is socially and culturally acceptable behavior for immigrant patients, and hence decreased treatment engagement [16]. Two studies using the Delphi process have recommended improvement of physician cultural competence as a step in improving substance use treatment in refugees and migrants in Europe [19,27]. Strategies proposed focus on the awareness of addiction service employees toward concepts that seem foreign or contradict their own convictions and involve focusing on each client as a unique case. Some recommended strategies include “understanding and acceptance of substance use as a coping strategy,” “cross- and transcultural competencies in attitude and reflection,” and “campaigns and interventions to reduce all forms of stigma, discrimination, and racism, targeting both service providers and migrants who use drugs themselves” [19,27].

Incorporating Social Support

Table 7 shows the pertinent findings of articles that discuss incorporating social support as a modality to improve treatment for immigrant populations [28,29].

**Table 7:** Pertinent findings of studies suggesting incorporating social support. aOR, adjusted odds ratio.

Study	Pertinent findings
Cox et al., 2022 [28]	Family-based academic success models appeared to slow the trajectory of substance use compared with national trends. Strengthening parental school involvement, positive parenting, youth personal agency, and peer affiliation likely buffered substance use escalation. The intervention leveraged Latino parents’ strong value on education to increase engagement and indirectly reduce risk behaviors.
Sanchez et al., 2020 [29]	Reduction in smoking after immigration was more likely among participants with higher pre-immigration social support (aOR: 1.87).

Some studies analyzed by this review have shown the advantage of incorporating the social support network of patients into their treatment as an effective strategy to improve care of immigrants with SUD [28,29]. A study by Sanchez et al. showed that a reduction in smoking after immigration was more likely among participants with higher pre-immigration social support (aOR: 1.87) [29]. A similar study on Latino adolescents in the United States suggested that a family-based academic success model appeared to slow the trajectory of substance use among the studied population compared with national trends [28].

Treating the Underlying Primary Mental Health Disorder

Table 8 shows the pertinent findings of articles that discuss treating underlying primary mental health disorders as a modality to improve treatment for immigrant populations [13,30].

**Table 8:** Pertinent findings of studies suggesting treating the underlying primary mental health disorder. AMA, against medical advice; CI, confidence interval; IIDEA, Integrated Intervention for Dual Problems and Early Action; OR, odds ratio.

Study	Pertinent findings
Alegría et al., 2019 [13]	For those with moderate-to-severe baseline substance use and mental health symptoms, IIDEA was effective in reducing substance use per the urine test result (OR: 0.25; 95% CI: 0.09-0.67; p=0.01). With regard to secondary outcomes, reduction in mental health symptoms was also greater among participants with moderate-to-severe mental health symptoms.
Sofer et al., 2018 [30]	44.6% of patients with schizophrenia, 44.4% of patients with diagnosed personality disorders, and 13.5% of patients with diagnosed depression did not complete their dual-diagnosis treatment program. The majority of them were AMA (χ^2^=15.940, p≤0.005).

With increased odds of concurrent mental health diagnoses among patients with SUD, studies identified by this review have highlighted the importance of treating these underlying mental health disorders to treat SUD in immigrant populations [13,30]. A study assessing early discharges from a dual-diagnosis treatment noted that the presence of primary mental health diagnoses played a statistically significant role in early discharges against medical advice (p<0.05). Of the total patients identified by this study, 44.6% of patients with schizophrenia, 44.4% of patients with diagnosed personality disorders, and 13.5% of patients with diagnosed depression did not complete their dual-diagnosis treatment program [30]. In terms of targeted treatment for potential dual-diagnosis candidates, IIDEA was found to be effective in reducing substance use (OR: 0.25, CI: 0.09-0.67, p=0.01), with greater reduction in mental health symptoms as a secondary outcome [13].

Other Interventions

Table 9 shows the pertinent findings of articles that discuss other modalities to improve treatment for immigrant populations [31].

**Table 9:** Pertinent findings of studies suggesting other interventions.

Study	Pertinent findings
Wada et al., 2017 [31]	Native English speakers reported higher awareness (92.5% vs. 88.3% vs. 50.0%, p<0.001) and ever use of e-cigarettes compared to foreign-born non-English-speaking participants, suggesting that cultural buffering may serve as a protective factor against e-cigarette use.

A study by Wada et al. identified cultural buffering as a protective factor against e-cigarette use among Spanish-speaking foreign-born individuals in the United States [30]. Per this study, native English speakers subjectively reported higher awareness (p<0.01) and ever use of e-cigarettes (p<0.001) compared with foreign-born and non-English-speaking participants [31].

**Figure 3:**
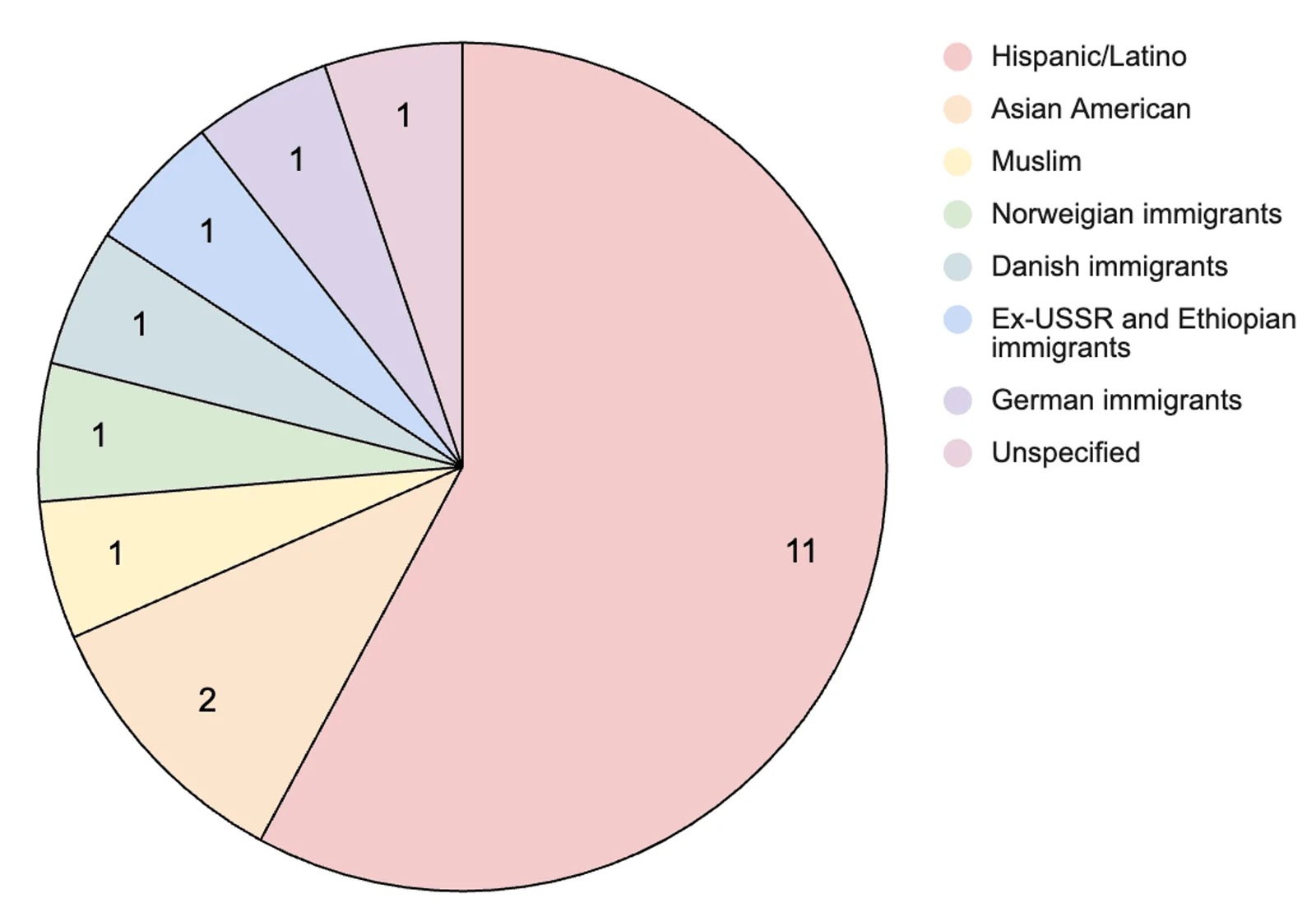
This pie chart summarizes the findings of the studies recorded in Table 1 and groups the populations studied by the papers identified in this review.

Figure 3 shows a pie chart of the groups of populations studied by the papers found in this scoping review of recent literature: Hispanic/Latino immigrants were studied by eleven articles, Asian American immigrants were studied by two articles, Muslim immigrants was studied by one article, three articles studied immigrants to the European countries of Norway, Germany, and Denmark, one article studied Ex-USSR and Ethiopian immigrants in Israel, and finally one article did not specify population background [13-31].

Discussion

Immigrant populations often possess distinct sociocultural identities and lived experiences that shape patterns of substance use and approach to treatment in ways that differ from native-born populations. These differences may reflect variations in cultural norms, migration-related stressors, and structural vulnerabilities. As a result, immigrants represent a socially and medically vulnerable group of people, necessitating that clinicians approach substance use assessment and treatment with a high degree of cultural competence.

The fundamental crux of this discussion is to understand that the immigrant identity differs significantly from that of those of native origin. As a result, immigrants’ experiences with substance use and its subsequent treatment can be vastly different from those of native origin. Based on the analysis of this systematic review, the biggest obstacle in engaging immigrants and refugees in SUD treatment remains the language gap between the patient and the healthcare provider. The language gap can result in patients underusing preventative healthcare options for substance use and non-compliance with their treatment course, thereby resulting in worse healthcare outcomes [32]. The authors of this review paper hypothesize that this might be explained by language discordance, which prevents immigrants from fully comprehending the diagnosis, its severity, and the course of treatment required for recovery. In fact, papers in this review have shown that appropriate patient-provider language concordance has increased patient retention and treatment completion [13,15]. One way to encourage language concordance in hospital settings is to train and use hospital-trained interpreters. Studies have shown higher satisfaction with hospital-trained interpreters than ad hoc (friends or family) or telephone interpreters [33]. A structural hurdle to the use of hospital-trained interpreters is that insurance does not cover the cost of interpreter services [32]. With heavy Medicaid reliance or uninsured rates among immigrants and refugees, coverage of interpretation services may encourage this population to seek treatment for SUD [21].

Despite the benefits of using an in-person interpreter, oftentimes, using bilingual staff is not enough to cover the diversity of patients’ native languages [19]. Evidence has also shown high accuracy and performance of language interpretation tools with commonly spoken languages, such as Spanish [34]. However, lower accuracy was noted with less common languages, such as Armenian and Farsi, suggesting that clinicians be cautious with less commonly spoken languages or those with fewer digital resources [34]. With regard to developing technology in the realm of language interpretation, ChatGPT was found to have improved semantic accuracy and fluency over other resources such as Google Translate [34]. However, ChatGPT continues to fall short of human-level performance for reasons such as prompt dependency and the potential to generate non-extant information, which has the risk of leading to serious negative outcomes in healthcare settings [34]. This information, however, highlights the potential for better-developed AI-based language models to serve as an interpretive service in a medical setting for non-English-speaking immigrants and refugees.

In concordance with the unique identity of immigrants, it is important to understand systemic stressors that impair this population’s ability to access care when needed. Some of the acculturative stress faced by this population can be attributed to risks of increased discrimination, family left abroad, fear of deportation, being female, family-cultural conflict, and ethnic enclave pressures [35]. Based on the findings of this review that acculturative stress is positively associated with alcohol and drug use severity, it can be hypothesized that substances are used by immigrant populations to cope with systemic stressors [22]. As a result, strategies to reduce SUD in these populations can include national-level legislative changes to discrimination and deportation laws to create protective factors for this community.

It is also important to recognize the role socioeconomic constraints play with regard to healthcare access. Many immigrant patients are unable to attend their medical appointments due to insufficient workplace accommodations, financial penalties for taking sick leave, lack of flexibility from employers, and incompatibility of clinic hours with patients’ work schedules [36]. However, data from this systematic review suggest significantly more quit attempts and increased likelihood of experiencing improvement when patients have been able to make their in-person physician appointments [13,20]. Therefore, interventions to help increase immigrants’ access to SUD treatment must include enforcing workplace regulations that guarantee protected time and flexibility for workers to attend medical appointments without financial penalty or risk of job loss. This is particularly critical given the widespread lack of workplace protections and prevalence of unethical employment practices in sectors employing undocumented immigrants, underscoring the need for policy reform that addresses these structural vulnerabilities.

Another area that warrants intervention is the reduction of sociocultural stigma surrounding alcohol/substance use disorder. There is no doubt that folks from different cultural backgrounds may treat addiction in different ways. As a result, stigma surrounding the topic may lead to immigrants not seeking treatment when they need it. This stigma can be self-inflicted toward oneself while dealing with substance use issues or socially motivated, which is a group-based form of disapproval or discrimination [36]. Existing literature suggests therapeutic interventions such as group-based acceptance and commitment therapy to reduce self-stigma [36]. This systematic review also found similar strategies, such as augmenting motivational interviewing with self-affirmation and utilizing culturally specific Juramentos, translating to mean oaths, as strategies to encourage immigrants to go through the course of SUD treatment [24,26]. Per existing literature, there is value in communicating positive stories of people with SUD to reduce social stigma [37]. This systematic review found that a strategy to destigmatize SUD across a religious social group is through the utilization of psychoeducation programs that combine religious principles and medical knowledge [23]. There remains added value in the social destigmatization of SUD, as the analysis of the papers in this review recommends incorporating the social support system of immigrant patients from an early age to curb substance use and also encourage quitting [28,29]. This intervention likely facilitates harm reduction on several levels of the immigrant experience and identity. One way in which social support plays a positive role in promoting treatment of SUD is through sociocultural stress buffering [31]. This review hypothesizes that incorporating social support likely operates through multiple pathways, including norm reinforcement, increased accountability, and reduction of structural barriers to care, all of which are particularly prominent in immigrant populations’ relationship with addiction treatment.

Another strategy identified by this review to “normalize” SUD treatment for a specific social group is to create interventions specific to that group of people. For example, this review identified anexos, juramentos, and the Vida PURA framework to be significantly successful in encouraging Latinx patients to seek SUD care and complete their treatment course [15,17,26]. While an existing limitation of this review is its inability to identify similar socioculturally specific interventions for other ethnic groups due to the distribution of populations studied by the papers of this review, highlighted in Figure 3, this review draws attention to the urgent need for intentional, equity-driven research that develops and evaluates culturally grounded SUD interventions across underrepresented immigrant communities.

Based on the analysis of this systematic review, physicians and healthcare practitioners have direct influence on their immigrant patients’ healthcare outcomes. As a result, some strategies have been identified to enhance the cultural competence of physicians and other healthcare providers. The strategies identified include “understanding and acceptance of substance use as a coping strategy,” “cross- and transcultural competencies in attitude and reflection,” and “campaigns and interventions to reduce all forms of stigma, discrimination, and racism, targeting both service providers and migrants who use drugs themselves” [19,27]. Fundamentally, these strategies serve to shift the focus away from the “culture” of the patient, as conceived by the nation-state or cultural circles, but to one’s own attitude. Ultimately, culturally competent addiction care requires clinicians to recognize the distinct identities and lived experiences of immigrant patients, to actively seek to understand each patient’s unique cultural context, and to deliver care that is both patient-centered and respectful, without reducing immigrant patients to “others.”

Another physician and healthcare practitioner intervention identified by this systematic review to improve SUD treatment outcomes in immigrant patients is to treat underlying primary mental health disorders if present [13,30]. The overlap between addiction disorders and mental health disorders is well studied, with some studies suggesting that >60% of patients with SUD have other comorbid psychiatric diagnoses [38,39]. In general, for patients with comorbid disorders, dual diagnosis settings have been shown to yield significant improvements in health outcomes, increased motivation, significant reduction in alcohol use, and increased remission rates [40,41]. As a result, it can be extrapolated that encouraging immigrant patients with SUD and underlying primary mental health disorders to receive dual diagnosis care would be a more effective approach. Furthermore, augmenting dual diagnosis care with certain therapeutic modalities such as IIDEA can be more effective in both the reduction of substance use and mental health symptoms [13].

The above-mentioned recommendations highlight the need for a systematic shift toward culturally responsive models of addiction care for immigrant populations. While existing evidence supports these strategies, further research is needed to evaluate their effectiveness across diverse settings and communities. Prioritizing such efforts will be essential to reducing disparities and advancing equitable, patient-centered care.

Limitations

This review is strengthened by a systematic, multi-database search strategy, independent multi-reviewer screening, and a structured PRISMA-guided methodology. However, as a systematic narrative review, our study carries certain limitations. A major limitation is that, as seen in Figure 3, the majority of articles analyzed in this review study the suggested strategies and interventions for Latinx populations. Other immigrant populations, ethnicities, and cultures make up just a minority of the data this review was able to synthesize and analyze. As a result, we call for further research to assess the effectiveness of culturally competent interventions on a diverse population of ethnic immigrant groups. Furthermore, the articles reviewed in this study contain data from multiple regions, including the United States, Europe, and the Middle East. As a result, a limitation of this paper is the lack of regional specificity of the suggested interventions. Finally, while several of the suggested interventions have been derived from data extracted from the articles analyzed in this review, others require further clinical-level research to estimate quantitative effectiveness and qualitative modifications as deemed appropriate. Furthermore, this being a narrative review, the reviewers did not perform any statistical analyses. This draws light to the need for meta-analysis to be performed by specialists in the appropriate fields for accurate quantitative data analysis.

## Conclusions

The findings of this review identify several interventions to provide culturally competent addiction treatment for immigrant patients. Given the prevalence of SUD in immigrant populations and the lack of treatment opportunities, a systemic shift toward culturally responsive models of care is vital for immigrant populations to have positive health outcomes. Therefore, physicians and healthcare providers must work with their patients and meet them where they are medically, socially, and culturally.
